# Association Between Serum Uric Acid Levels and Benign Paroxysmal Positional Vertigo: A Systematic Review and Meta-Analysis of Observational Studies

**DOI:** 10.3389/fneur.2019.00091

**Published:** 2019-02-15

**Authors:** Xinglong Yang, Baiyuan Yang, Mengjun Wu, Fang Wang, Xiaodong Huang, Kelu Li, Zhiwei Mao, Zhong Xu, Hui Ren

**Affiliations:** ^1^Department of Geriatric Neurology, First Affiliated Hospital of Kunming Medical University, Kunming, China; ^2^Department of Neurology, Seventh People's Hospital of Chengdu, Chengdu, China; ^3^Department of Anesthesiology, Chengdu Women and Children's Central Hospital, Chengdu, China

**Keywords:** benign paroxysmal positional vertigo, peripheral vertigo, otolithiasis, serum uric acid, meta analysis

## Abstract

**Objective:** The objective of the present study was to meta-analyze relevant literature to gain a comprehensive understanding of the potential relationship between serum uric acid levels and risk of benign paroxysmal positional vertigo (BPPV).

**Methods:** The databases of PubMed, Web of Science, Embase, Chinese National Knowledge Infrastructure, Wanfang, and SinoMed were systematically searched for observational case-control studies of the association between BPPV and serum uric acid levels published up to October 2018. Data from eligible studies were meta-analyzed using Stata 12.0.

**Results:** A total of 12 studies were included in the analysis. There was a strong tendency for serum uric acid levels to be associated with risk of BPPV among studies conducted in China (OR 0.69, 95%CI 0.01–1.40, *p* = 0.053), but not among studies outside China (OR 1.07, 95%CI 1.08–3.22, *p* = 0.33). Across all studies, serum uric acid level was significantly higher among individuals with BPPV than among controls (OR 0.78, 95%CI 0.15–1.41, *p* = 0.015), yet it did not independently predict risk of the disorder (OR 1.003, 95%CI 0.995–1.012, *p* = 0.471).

**Conclusion:** The available evidence suggests that BPPV is associated with elevated levels of serum uric acid, but these levels may not be an independent risk factor of BPPV.

## Introduction

Benign paroxysmal positional vertigo (BPPV) is one of the most common causes of peripheral vestibular vertigo encountered in neuro-otology clinics (1). Its main characteristic is short, recurring episodes of vertigo induced when the head adopts certain positions. This occurs because the otoconia are dislodged and enters one or more semicircular canals, which disturbs endolymphatic flow and results in vertigo. BPPV can be diagnosed according to recommended criteria (2) and repositioning of the head can treat BPPV effectively (3), but preventive treatment and monitoring would be improved with better understanding of the risk factors for BPPV. Old age, vitamin D deficiency, and osteoporosis have been cited as risk factors (4).

Some studies have also implicated elevated serum levels of uric acid (5, 6), but other work has not replicated these findings (7–9). Within China, studies of this question have reported inconsistent findings (10–17). It is important to clarify this potential association because humans are susceptible to accumulation of uric acid in serum, which is produced through the metabolism of endogenous and exogenous purines (18, 19). Humans lack the enzyme uricase, which in other mammals metabolizes uric acid into the more soluble allantoin (20). As a result, serum uric acid levels can rise if uric acid production is excessive and/or if uric acid is cleared too slowly. In addition, some work suggests that increased serum uric acid level might be an independent risk factor for hypertension (21), cardiovascular events (22), metabolic syndrome (23), ischemic stroke (24), as well as BPPV (6).

Here we meta-analyzed the relevant literature to gain a comprehensive understanding of the potential relationship between serum uric acid levels and risk of BPPV.

## Methods

### Search Strategy

The databases of PubMed, Web of Science, Chinese National Knowledge Infrastructure, Wanfang, SinoMed, and Embase were systematically searched for eligible studies published up to October 2018. The search terms were “benign paroxysmal positional vertigo” and “serum uric acid.” No language or date restrictions were applied.

### Study Selection Criteria

To be included in the meta-analysis, studies had to (1) be observational studies with a case-control design analyzing the association between BPPV and serum uric acid; (2) rely on diagnosis based on a typical history of brief attacks of positional vertigo and confirmed by a positive Dix-Hallpike positional maneuver (posterior canal BPPV), or by the presence of a purely horizontal paroxysmal nystagmus provoked during the supine roll test (horizontal semicircular canal BPPV), in which the head is turned by about 90° to each side while the individual is supine; and (3) report the mean levels of serum uric acid for case and control groups, or sufficient data to calculate these values. If more than one study evaluated the same cohort, only the study with the most complete data was included.

Studies were excluded if they (1) were editorials, reviews, case reports, letters without original data, commentaries or critiques; or (2) they failed to report adequate data to determine mean serum uric acid levels in the case and control groups.

### Data Extraction

Two investigators (XL Yang and BY yang) independently searched the literature databases and extracted data. Inconsistencies were resolved through consultation with a third author (H Ren). The following data were collected from studies included in the review (Table 1): surname of the first author, country of study cohort, year of publication, serum uric acid level as well as odds ratios (ORs) and associated 95% confidence intervals (CIs) for multiple factor analyses.

**Table 1:** Main characteristics of studies on benign paroxysmal positional vertigo (BPPV) and serum uric acid included in this review.

**References**	**Country**	***n***	**Serum uric acid level (μmol/L)**		**Multiple logistic regression**	
			**BPPV group**	**Control group**	**OR**	**95%CI**
Ziavra and Bronstein (7)	UK	20/20	290.35 ± 96.11	273.4 ± 88.27	–	–
Jeong and Kim (9)	South Korea	168/194	285.6 ± 77.35	315.35 ± 83.3	0.8	0.7–1.0
Celikbilek et al. (6)	Turkey	50/40	288.58 ± 23.8	214.2 ± 18.6	3.35	1.87–5.99
Xu et al. (14)	China	150/89	297 ± 30.94	201 ± 20.98	3.58	1.67–5.68
Zhai et al. (11)	China	80/80	375.48 ± 79.746	331.56 ± 62.262	1.009	1.004–1.014
Zhu et al. (17)	China	102/85	317.7 ± 78.4	289.2 ± 71.8	1.005	1.001–1.009
Si (16)	China	50/50	888.34 ± 245.74	343.9 ± 124.36	–	
Yuan et al. (8)	China	240/72	279.0 ± 84.7	331.0 ± 82.7	0.997	0.992–1.001
Dong et al. (12)	China	110/50	333.6 ± 84.78	304.3 ± 76.55	0.995	0.990–1.000
Lu et al. (13)	China	60/119	321 ± 98	322 ± 92	1.273	0.554–2.926
Wei et al. (15)	China	102/100	361.174 ± 84.335	298.626 ± 87.861	–	–
Xia et al. (10)	China	80/80	343.52 ± 72.83	290.36 ± 84.23	1.010	1.004–1.016

### Quality Assessment of Studies

Two reviewers (XL Yang and BY Yang) independently assessed the quality of studies using the Newcastle-Ottawa quality assessment scale. Discrepancies were resolved through consultation with a third author (H Ren). Scores≥6 indicated that a study was of high quality (25) (Table 2).

**Table 2:** Quality assessment.

**References**	**Score on dimensions**			**Total score**
	**Selection**	**Exposure**	**Comparability**	
Ziavra and Bronstein (7)	3	2	1	6
Jeong and Kim (9)	3	2	2	7
Celikbilek et al. (6)	2	2	2	6
Xu et al. (14)	3	2	2	7
Zhai et al. (11)	3	2	2	7
Zhu et al. (17)	3	2	2	7
Si (16)	2	2	1	5
Yuan et al. (8)	3	2	2	7
Dong et al. (12)	2	2	2	6
Lu et al. (13)	2	2	2	6
Wei et al. (15)	3	2	1	6
Xia et al. (10)	2	2	2	6

### Statistical Analysis

I^2^ was calculated to evaluate heterogeneity across studies. I^2^ < 25% indicated homogeneity; 25% ≤ I^2^ < 50%, low heterogeneity; 50% ≤ I^2^ < 75%, moderate heterogeneity; and I^2^ ≥ 75%, substantial heterogeneity (26). Data were meta-analyzed using a fixed-effect model if they were homogeneous or of low heterogeneity, or using a random-effect model if they showed moderate or substantial heterogeneity (27). All meta-analyses were conducted using Stata 12.0 (StataCorp, USA). We compared the serum uric acid levels between individuals with BPPV and controls in terms of ORs and 95%CIs. We pooled ORs for multiple-factor analysis. Significance was defined as *p* < 0.05. Risk of publication bias was assessed using Egger's and/or Begg's tests (27, 28).

## Results

### Literature Search and Included Studies

**Figure 1:**
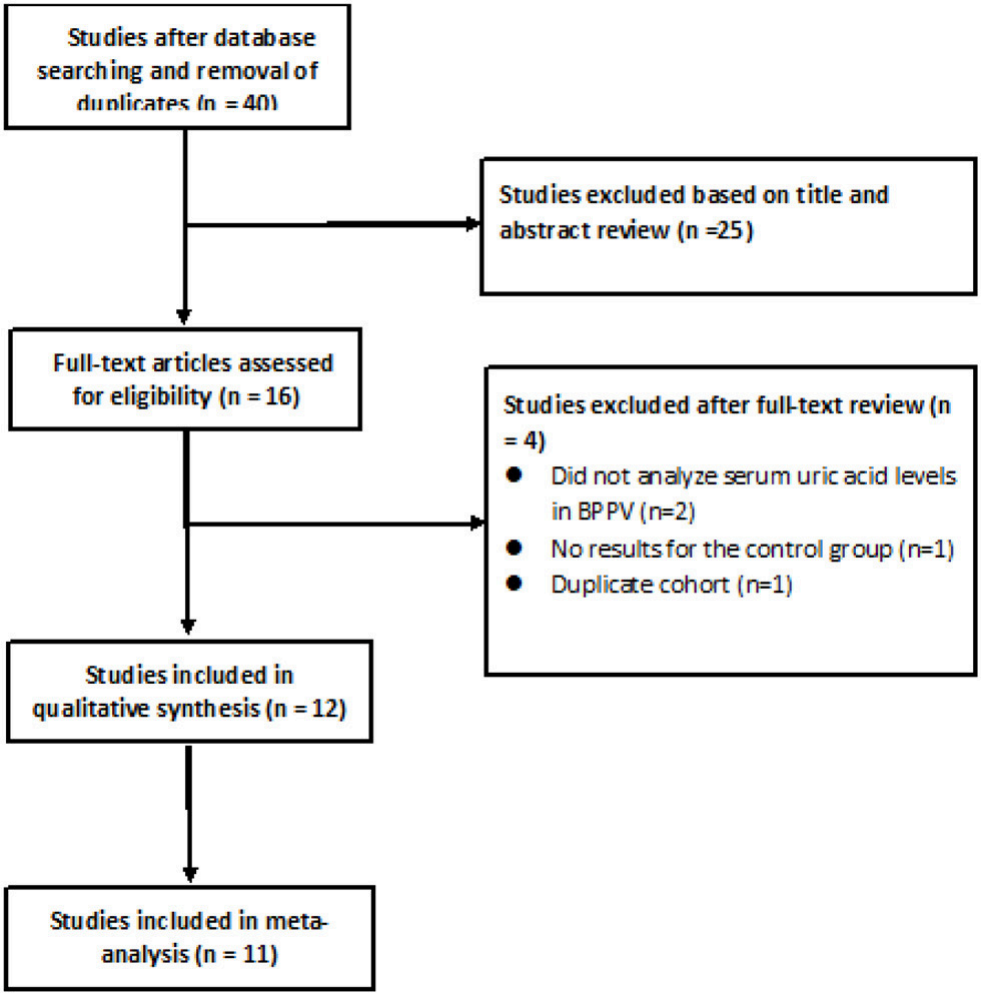
Flow diagram of study selection.

After searching the above databases and removing duplicates, 40 potentially eligible articles were identified, of which 25 were excluded based on the titles and abstracts (Figure 1). Of the remaining 15 studies read in full, 2 were excluded because they did not analyze serum uric acid levels in individuals with BPPV, 1 study was excluded because it did not report results for the control group, 1 study was excluded because its cohort was identical to that of another study, and 1 study (16) was excluded because of a low Newcastle-Ottawa score.

Of the remaining 12 studies, involving 1,162 BPPV cases and 929 controls, 8 were conducted in China and 3 outside (Table 1). Nine studies used multiple logistic analysis to investigate whether serum uric acid levels are associated with BPPV.

### Comparison of Serum Uric Acid Levels Between Cases and Controls

**Figure 2:**
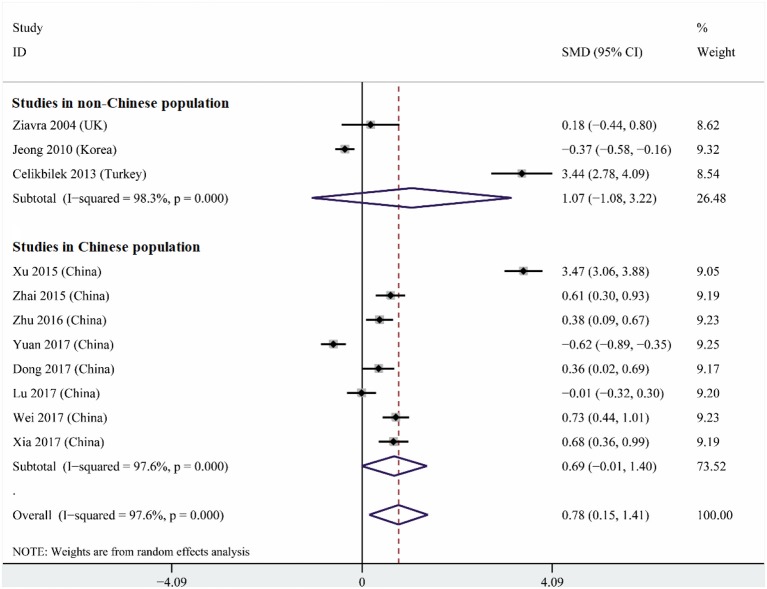
Forest plot of serum uric acid levels in the BPPV and control groups across all studies, in the subset of studies conducted within China or in the subset conducted outside China. The x-axis shows the 95% confidence interval. SMD, standard mean difference.

**Figure 3:**
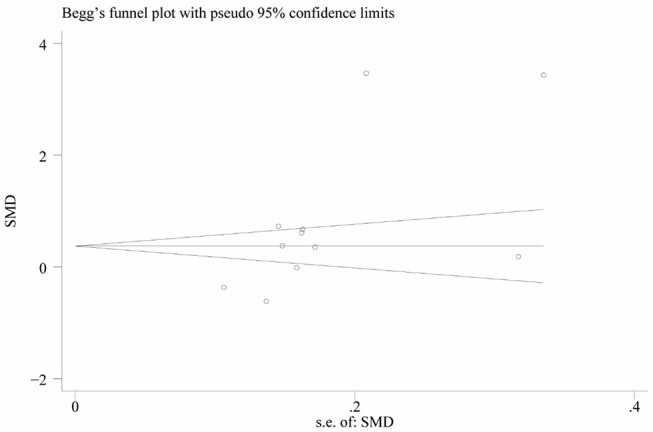
Funnel plot of standard mean difference (SMD) in serum uric acid level between the BPPV and control groups.

**Figure 4:**
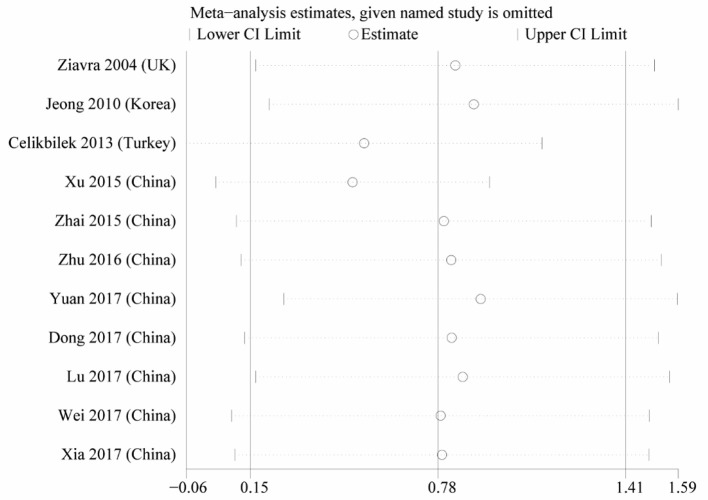
Sensitivity analysis of the studies in serum uric acid level between the BPPV and control groups.

Heterogeneity was high among the 11 studies (I^2^ = 97.6%, *p* < 0.001), so data were meta-analyzed using a random-effect model. Serum uric acid levels were significantly higher in individuals with BPPV than in controls (OR 0.78, 95%CI 0.15 to 1.41, *p* = 0.015; Figure 2). These results were not significantly altered when any one of the 11 studies was removed (Figure 3). The funnel plot was visually symmetrical, suggesting no significant publication bias (Figure 4); a similar conclusion was suggested by Egger's test (*p* = 0.827) and Begg's test (0.702).

Subgroup analysis based on study location indicated that a strong tendency for serum uric levels to be associated with risk of BPPV existed in China (OR 0.69, 95%CI 0.01–1.40, *p* = 0.053), but not outside (OR 1.07, 95%CI 1.08–3.22, *p* = 0.33; Figure 2). These results were obtained using a random-effect meta-analysis model because the data showed high heterogeneity.

### Meta-Analysis of Multiple Logistic Analyses to Identify Risk Factors for BPPV

**Figure 5:**
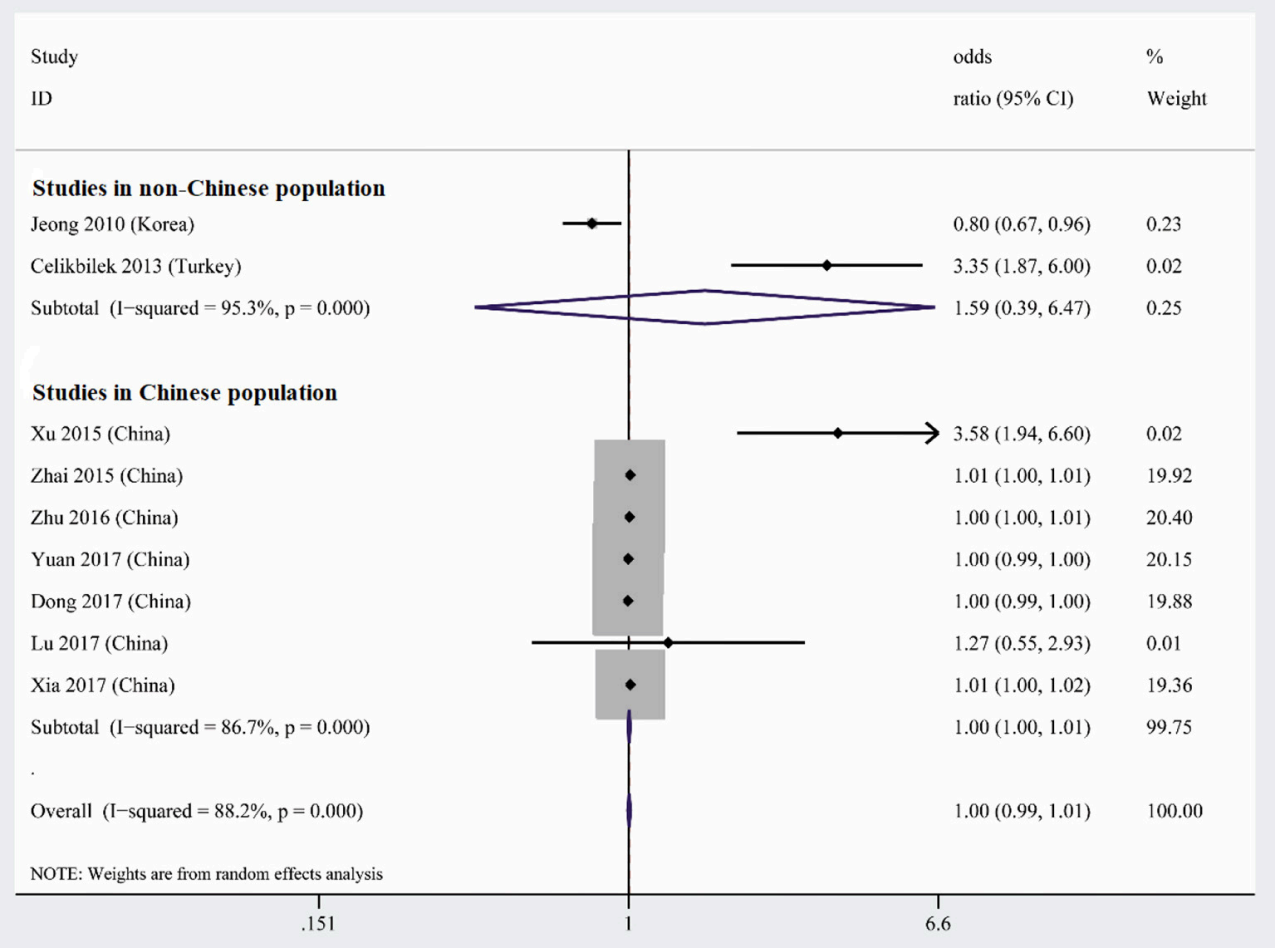
Forest plot of serum uric acid level as an independent risk factor for BPPV across all studies, in the subset of studies conducted within China or in the subset conducted outside China. The x-axis shows the 95% confidence interval.

**Figure 6:**
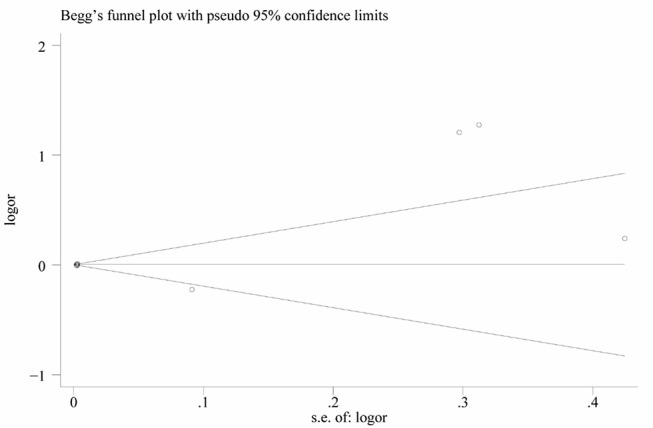
Funnel plot of serum uric acid level as an independent risk factor for BPPV.

Nine studies performed multiple logistic analyses to identify risk factors for BPPV (Table 1). These results were meta-analyzed using a random-effect model because of high heterogeneity in the data. The pooled results indicate that serum uric acid level is not an independent risk factor (OR 1.003, 95%CI 0.995–1.012, *p* = 0.471; Figure 5), and this was also the case for subsets of studies within or outside China. The funnel plot was visually symmetrical (Figure 6), and Egger's and Begg's tests were associated with *p* > 0.05, suggesting no significant risk of publication bias.

## Discussion

To the best of our knowledge, this is the first meta-analysis to evaluate the potential association between BPPV and serum uric acid level. The available evidence appears to indicate that serum uric acid level is elevated in BPPV, but the level may not be an independent risk factor for the disorder.

In other contexts, elevated serum uric acid levels show complex relationships with disease. Elevated levels have been associated with cardiovascular disease and stroke (29), but they may exert protective anti-oxidant effects against neurodegenerative diseases such as Parkinson's disease (30). Most studies in our meta-analysis found a higher serum uric acid level in individuals with BPPV than in controls. This was first shown in an African population (5), and supported by several other studies in other countries (6, 10–12, 14–17). On the other hand, some studies reported similar serum uric levels in BPPV and control groups (7, 13), while two studies from Asia reported lower levels in BPPV (8, 9). Our subgroup analysis suggests that some of the discrepancy in studies of the association between serum uric acid level and BPPV relates to ethnicity and/or geography: we found a strong tendency toward an association within China but not outside. These results suggest that further work should examine genetic and environmental factors that may mediate an association between serum uric acid levels and BPPV.

While most studies in our meta-analysis linked BPPV with elevated serum uric acid levels, it is another question whether such levels can serve as an independent predictor of the disorder. One study in Turkey reported that elevated levels are an independent risk factor of the disorder (6), and four studies from China came to a similar conclusion (10, 14, 17, 31). However, several other studies reported that elevated uric acid levels are not an independent risk factor, as we found when we meta-analyzed all studies or subsets of studies conducted inside or outside China. Evidence suggests that this conclusion is valid for younger and older populations with BPPV (13).

Although our meta-analysis argues against serum uric acid levels as an independent risk factor for BPPV, serum uric acid may still contribute to the disorder. One study found a positive association between gout and peripheral vertigo, leading those authors to speculate that build-up of purine crystal deposits within the semicircular canals may trigger BPPV in individuals with gout (32). It is also possible that elevated serum uric acid levels can trigger inflammation of the gelatinous matrix to which otoconia are connected (33, 34); uric acid can promote the release of inflammatory mediators that induce production of damaging reactive oxygen species (ROS). Through a similar inflammatory mechanism, elevated serum uric acid levels may trigger production of ROS that damage the vasculature (35, 36), compromising blood supply to the inner ear. Consistent with these processes, a study based on native thiol/disulfide (SH/SS) homeostasis as a novel indicator of oxidative stress suggested that oxidative stress contribute to BPPV through both calcium metabolism and the direct toxic effects of free oxygen radicals (37). Another study found that frog saccular otoconia dissolved completely within 1 day in inner lymphatic fluid when the Ca^2+^ concentration was physiologically low (20 μmol/L), but they required hundreds of hours to dissolve when the Ca^2+^ concentration was 200 μmol/L and did not dissolve when the concentration was 500 μmol/L (38). For example, an increase of calcium resorption in patient with osteoporosis/osteopenia might generate an increased concentration of free calcium in the endolymph and reduce its capacity to dissolve the dislodged otoconia (39). Furthermore, if uric acid enters the lymphatic fluid, reduces pH will preventing dissolution of otolith fragments. Thus, either the raised calcium levels induced by oxidative stress or reduced pH in the endolymph will destroy the normal equilibrium between otolith formation and dissolution, potentially leading to BPPV. Consistent with these considerations, allopurinol lowers serum uric acid levels and restores endothelial function to nearly normal levels (40).

The conclusions from our study should be interpreted with caution in light of several limitations. The risk of publication bias always exists, although we did search a range of international and Chinese databases without language constraints, and Egger's and Begg's tests suggested no significant risk of such bias. Although 12 studies were included in our review, the total sample was only 1,162 BPPV cases and 929 controls, and most of the studies were conducted on Chinese cohorts. Future studies should seek to verify our results in a broader range of populations. Despite the predominance of one country in our sample, our data showed substantial heterogeneity. This may reduce the reliability of our results, although we did not see substantial effects in sensitivity analyses in which we systematically omitted studies one at a time. Some of the included studies did not describe clearly whether subjects used allopurinol or diuretics or what their nutritional and physical exercise habits were, all of which can affect serum uric acid levels.

Even with these limitations, our meta-analysis provides an overall view of available evidence suggesting that BPPV is associated with elevated serum uric acid levels but may not be an independent risk factor for the disorder. These results should be verified and extended in large, well-designed studies involving multiple ethnic groups.

## Author Contributions

All authors designed the study. BY conducted the analysis with advice from all authors, especially XY, HR, and BY authored the manuscript with input and revisions provided by all authors. Each author has given final approval of the manuscript's publication and agrees to be accountable for all aspects of the work. ZM and ZX contributed to the revision of the article.

### Conflict of Interest Statement

The authors declare that the research was conducted in the absence of any commercial or financial relationships that could be construed as a potential conflict of interest.

## References

[1] Hornibrook J. Benign Paroxysmal Positional Vertigo (BPPV): history, pathophysiology, office treatment and future directions. Int J Otolaryngol. (2011) 2011:835671. 10.1155/2011/835671 21808648 PMC3144715

[2] von Brevern M Bertholon P Brandt T Fife T Imai T Nuti D. Benign paroxysmal positional vertigo: diagnostic criteria. J Vestib Res. (2015) 25:105–17. 10.3233/VES-150553 26756126

[3] Perez-Vazquez P Franco-Gutierrez V Soto-Varela A Amor-Dorado JC Martin-Sanz E Oliva-Dominguez M. Practice guidelines for the diagnosis and management of benign paroxysmal positional vertigo otoneurology committee of spanish otorhinolaryngology and head and neck surgery consensus document. Acta Otorrinolaringol Espanola (2018) 69:345–66. 10.1016/j.otoeng.2018.10.002 28826856

[4] Chen CC Cho HS Lee HH Hu CJ. Efficacy of repositioning therapy in patients with benign paroxysmal positional vertigo and preexisting central neurologic disorders. Front Neurol. (2018) 9:486. 10.3389/fneur.2018.00486 30013505 PMC6037198

[5] Adam AM Benign positional vertigo as a clinical manifestation of hyperuricemia - a recent discovery. J Neurol Sci. (2001) 187(Suppl. 1):222.

[6] Celikbilek A Gencer ZK Saydam L Zararsiz G Tanik N Ozkiris M. Serum uric acid levels correlate with benign paroxysmal positional vertigo. Eur J Neurol. (2014) 21:79–85. 10.1111/ene.12248 23952220

[7] Ziavra NV Bronstein AM. Is uric acid implicated in benign paroxysmal positional vertigo? J Neurol. (2004) 251:115. 10.1007/s00415-004-0277-7 14999502

[8] Yuan J Dai J Li WA Hu W. Factors Associated with benign paroxysmal positional vertigo: a chinese case-control study. Med Sci Monit. (2017) 23:3885–9. 10.12659/MSM.905716 28800356 PMC5565235

[9] Jeong SH Kim JS The effect of serum uric acid in generating idiopathic benign paroxysmal positional vertigo. Res Vestib Sci. (2010) 9:27–31.

[10] Xia F Wang N Wang Y Liang L Liu M Li J The relationship between benign paroxysmal positional Vertigo and cerebrovascular disease in the eIderly. Chin J Geriatr. (2017) 36:1087–91. 10.3760/cma.j.issn.0254-9026.2017.10.008

[11] Zhai G She Z Shao G Hu L Relationship between uric acid and benign paroxysmal positional vertigo. J Neurosci Mental Health (2015) 15:177–9. 10.3969/j.issn.1009-6574.2015.02.020

[12] Dong L Wang Z Zhang W Bao L Cui G Ye X The correlation between serum uric acid level and benign paroxysmal position vertigo. Chin J Pract Nervous Dis. (2017) 20:21–3. 10.3969/j.issn.1673-5110.2017.21.005

[13] Lu L Wang W Fan C Li J Risk factors associated with benign paroxysmal positional vertigo–a case-control study. J Nantong Univ. (2017) 37:442–4. 10.16424/j.cnki.cn32-1807/r.2017.05.012

[14] Xu M Chen W Liu L Wang F Ling Y Huang Z Study on the correlation between uric acid level and benign paroxysmal positional vertigo. J Xiangnan Univ. (2015) 17:11–3. 10.16500/j.cnki.1673-498x.2015.03.004

[15] Wei S Liu J Liu Z Plasma homocysteine and uric acid levels in young patients with benign paroxysmal positional vertigo and their correlations. Chin J Ophthalmol Otorhinolaryngol (2017) 17:126–751. 10.14166/j.issn.1671-2420.2017.02.014

[16] Si J Analysis of serum uric acid level in patients with benign paroxysmal positional vertigo. Qinghai Med J. (2016) 46:54–5.

[17] Zhu X Sima G Dai L Ji W Relationship between benign paroxysmal positional vertigo and serum uric acid level in elderly patients. Chin Arch Otolaryngol Head Neck Surg. (2016) 23:696–9. 10.16066/j.1672-7002.2016.12.004

[18] Maiuolo J Oppedisano F Gratteri S Muscoli C Mollace V. Regulation of uric acid metabolism and excretion. Int J Cardiol. (2016) 213:8–14. 10.1016/j.ijcard.2015.08.109 26316329

[19] Pacher P Nivorozhkin A Szabo C. Therapeutic effects of xanthine oxidase inhibitors: renaissance half a century after the discovery of allopurinol. Pharmacol Rev. (2006) 58:87–114. 10.1124/pr.58.1.6 16507884 PMC2233605

[20] Kratzer JT Lanaspa MA Murphy MN Cicerchi C Graves CL Tipton PA. Evolutionary history and metabolic insights of ancient mammalian uricases. Proc Natl Acad Sci USA. (2014) 111:3763–8. 10.1073/pnas.1320393111 24550457 PMC3956161

[21] Lee SW Kim HC Nam C Lee HY Ahn SV Oh YA Age-differential association between serum uric acid and incident hypertension. Hypertens Res. (2018) 9:27–31. 10.1038/s41440-018-0168-4 30559402

[22] Tian TT Li H Chen SJ Wang Q Tian QW Zhang BB. Serum uric acid as an independent risk factor for the presence and severity of early-onset coronary artery disease: a case-control study. Dis Mark. (2018) 2018:1236837. 10.1155/2018/1236837 30425752 PMC6218741

[23] Cicero AFG Fogacci F Giovannini M Grandi E Rosticci M D'Addato S. Serum uric acid predicts incident metabolic syndrome in the elderly in an analysis of the Brisighella Heart Study. Sci Rep. (2018) 8:11529. 10.1038/s41598-018-29955-w 30068918 PMC6070523

[24] Arevalo-Lorido JC Carretero-Gomez J Robles Perez-Monteoliva NR Association between serum uric acid and carotid disease in patients with atherosclerotic acute ischemic stroke. Vascular (2018) 2018:1708538118797551 10.1177/1708538118797551 30205779

[25] Stang A. Critical evaluation of the Newcastle-Ottawa scale for the assessment of the quality of nonrandomized studies in meta-analyses. Eur J Epidemiol. (2010) 25:603–5. 10.1007/s10654-010-9491-z 20652370

[26] Higgins JP Thompson SG Deeks JJ Altman DG. Measuring inconsistency in meta-analyses. BMJ (2003) 327:557–60. 10.1136/bmj.327.7414.557 12958120 PMC192859

[27] Zhang TS Zhong WZ Applied Methodology for Evidence-based Medicine. (2012). p. 166–75.

[28] Egger M Smith GD Phillips AN. Meta-analysis: principles and procedures. BMJ (1997) 315:1533–7. 10.1136/bmj.315.7121.1533 9432252 PMC2127925

[29] Benn CL Dua P Gurrell R Loudon P Pike A Storer RI. Physiology of hyperuricemia and urate-lowering treatments. Front Med. (2018) 5:160. 10.3389/fmed.2018.00160 29904633 PMC5990632

[30] Gonzalez-Aramburu I Sanchez-Juan P Sierra M Fernandez-Juan E Sanchez-Quintana C Berciano J. Serum uric acid and risk of dementia in Parkinson's disease. Parkins Rel Disord. (2014) 20:637–9. 10.1016/j.parkreldis.2014.02.023 24637121

[31] Siu YP Leung KT Tong MK Kwan TH. Use of allopurinol in slowing the progression of renal disease through its ability to lower serum uric acid level. Am J Kidney Dis. (2006) 47:51–9. 10.1053/j.ajkd.2005.10.006 16377385

[32] Lin YT Lin HW Huang YC Ho WT Li YC Chen TJ. Association between gout and vertigo in a Taiwanese population. J Clin Neurosci. (2013) 20:857–61. 10.1016/j.jocn.2012.05.036 23394876

[33] Lins U Farina M Kurc M Riordan G Thalmann R Thalmann I. The otoconia of the guinea pig utricle: internal structure, surface exposure, and interactions with the filament matrix. J Struct Biol. (2000) 131:67–78. 10.1006/jsbi.2000.4260 10945971

[34] Chen DP Wong CK Tam LS Li EK Lam CW. Activation of human fibroblast-like synoviocytes by uric acid crystals in rheumatoid arthritis. Cell Mol Immunol. (2011) 8:469–78. 10.1038/cmi.2011.35 21946433 PMC4012929

[35] Chao HH Liu JC Lin JW Chen CH Wu CH Cheng TH. Uric acid stimulates endothelin-1 gene expression associated with NADPH oxidase in human aortic smooth muscle cells. Acta Pharmacol Sin. (2008) 29:1301–12. 10.1111/j.1745-7254.2008.00877.x 18954524

[36] Khosla UM Zharikov S Finch JL Nakagawa T Roncal C Mu W. Hyperuricemia induces endothelial dysfunction. Kidney Int. (2005) 67:1739–42. 10.1111/j.1523-1755.2005.00273.x 15840020

[37] Sahin E Deveci I Dinc ME Ozker BY Bicer C Erel O. Oxidative status in patients with benign paroxysmal positional vertigo. J Int Adv Otol. (2018) 14:299–303. 10.5152/iao.2018.4756 30256204 PMC6354457

[38] Zucca G Valli S Valli P Perin P Mira E. Why do benign paroxysmal positional vertigo episodes recover spontaneously? J Vestibul Res. (1998) 8:325–9. 10.1016/S0957-4271(97)00080-3 9652482

[39] Vibert D Kompis M Hausler R. Benign paroxysmal positional vertigo in older women may be related to osteoporosis and osteopenia. Ann Otol Rhinol Laryngol. (2003) 112:885–9. 10.1177/000348940311201010 14587980

[40] Butler R Morris AD Belch JJ Hill A Struthers AD. Allopurinol normalizes endothelial dysfunction in type 2 diabetics with mild hypertension. Hypertension (2000) 35:746–51. 10.1161/01.HYP.35.3.746 10720589

